# Bridging the gap between evidence and implementation for healthy ageing

**DOI:** 10.1016/j.lanwpc.2023.100834

**Published:** 2023-06-28

**Authors:** The Lancet Regional Health – Western Pacific

Advances in society have led to longer lives, but the quality of those extra years is not guaranteed to be a positive experience. We are currently amid the UN Decade of Healthy Ageing (2021–30), which aims to transform opportunities for everyone to have better wellbeing in their later years. The Decade focuses on four action areas to address the health and social challenges that existing societal infrastructures face due to the shifting demographics in an ageing population. The movement calls for action to combat ageism, create age-friendly environments, provide integrated care, and provide access to long-term care and support when needed. These actions summarise what ageing populations need: a systematic shift in perspective that extends beyond the health-care system and is embedded throughout society.

For a systematic change in health care, the division of microlevel, mesolevel, and macrolevel systems theory can be applied to patients and health-care providers, health organisations, and health policy, respectively. However, the magnitude of change required to encourage healthy ageing would reframe these levels: microlevel focuses would centre on the ageing individuals and their private network, including carers. Mesolevel structures would expand to include community factors such as civic attitudes, public infrastructures, and health organisations. Finally, the macrolevel would expand to include policies from branches of government beyond health as well as other public and private entities. To enact change, evidence must be generated for transformations in each of these domains.

In this issue of the journal, we present a special collection on healthy ageing in the Western Pacific region, home to over 240 million people over the age of 65 years—more than any other region in the world. Viewpoints by Lee and colleagues and Wang and Yu discuss the implementation of social prescribing—an approach that addresses the individual’s social and cognitive health while requiring mesolevel input by health-care providers and the provision of community assets. In Singapore, social prescribing was implemented in July, 2019. However, a paucity of evidence required implementers to use an iterative approach to maximise the impact of change. To address the inadequate implementation evidence for mental health social prescribing, Wang and Yu propose three elements to underscore the solutions: stakeholders, contextual factors, and outcome measures. The collection also examines digital technologies for social and physical health in the context of information consolidation and health access, gamification, and personified artificial intelligence to update infrastructure at the mesolevel for a follow-on effect to microlevel stakeholders. These Articles highlight the potential and feasibility of change using existing knowledge of intervention performance and technologies. However, they again add credence to the primary problem—a paucity of evidence to enact change.

At present, research in healthy ageing is primarily focused on evidence generation. Many publications report the performance of interventions within a confined silo of strict research parameters but lack the real-world situations and conditions that would exclude many individuals as participants. For example, oncology research in older adults often excludes individuals who are frail with comorbidities but would otherwise account for a substantial portion of the real-world demographic. Implementation studies are the essential next step to translate knowledge into practice by assessing the true potential and capacity for the scale-up of innovations. However, evidence to support implementation is often weak, lacks generalisability, or is even wholly absent for many underserved groups, as highlighted in this special collection. Implementation studies that investigate overlooked outcomes, including acceptance and adoption, sustainability, and cost of implementation in microlevel and mesolevel structures, are needed to drive policy change at the macrolevel.

Furthermore, the Western Pacific region’s social, cultural, and economic diversity dictates that these studies cannot be a one size fits all for effective knowledge translation. Implementation research needs to be considered separately in each community and country based on the attitudes of all involved, the capacity of care providers, and the availability of assets. However, the novelty of implementation science is reflected in the insufficient number of experts, and in low-income and middle-income countries (LMICs), resource limitation would pose substantial challenges to collecting such evidence. In these instances, evidence from high-income countries could be applied to LMICs, but iterative approaches must be used to account for the differences in setting for maximal impact.

Our society is rapidly ageing. We are not lacking in innovations that could change the quality of life for older people, but we must move towards building a foundation of solid evidence from which we can leap into meaningful change. We need to strengthen the research continuum at all systematic levels to transform evidence to practice for the much-needed shifts in perspective. Only then can we see systematic changes that will provide every individual with equitable opportunities for healthy ageing.

